# Correction: Zhu et al. Biodegradable and pH Sensitive Peptide Based Hydrogel as Controlled Release System for Antibacterial Wound Dressing Application. *Molecules* 2018, *23*, 3383

**DOI:** 10.3390/molecules27196682

**Published:** 2022-10-08

**Authors:** Jie Zhu, Hua Han, Ting-Ting Ye, Fa-Xue Li, Xue-Li Wang, Jian-Yong Yu, De-Qun Wu

**Affiliations:** 1 Key Laboratory of Textile Science and Technology, Ministry of Education, College of Textiles, Donghua University, Songjiang District, Shanghai 201620, China; 2 Modern Textile Institute, Donghua University, Changning District, Shanghai 200051, China

During the course of a review of our publication, we found two errors in Figure 4b and Figure 9. We wish to make the following corrections to this paper [1]. We have inserted SEM and H&E images mistakenly, but the results and conclusions of the paper are not affected. We have provided the correct figures below.

All co-authors agree with the content of this correction and we would like to apologize for any inconvenience caused to the readers by these changes.

**Figure 4:**
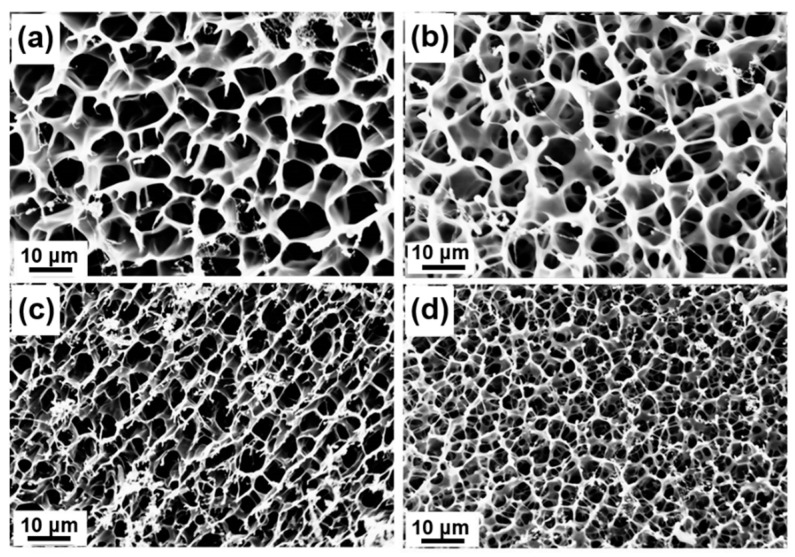
SEM images of homogeneous peptide-based bis-acrylate/AAc hydrogels before biodegradation: (**a**) Gel-1; (**b**) Gel-2; (**c**) Gel-3; (**d**) Gel-4. With the increasing of peptide-based bis-acrylate contents, the pore size of the hydrogels would decrease.

**Figure 9:**
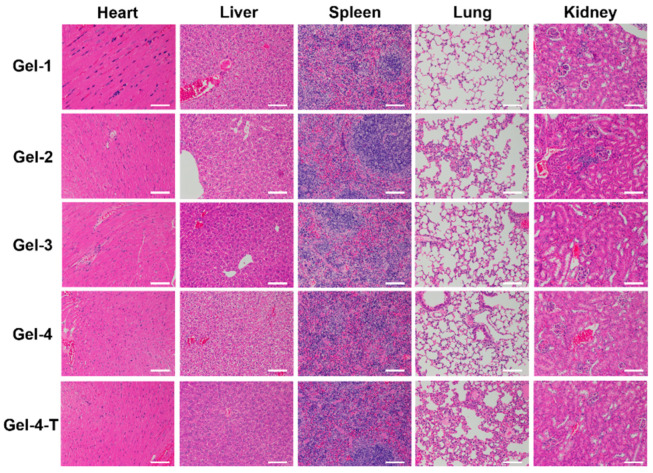
In vivo toxicity assessment of hydrogels. Hematoxylin-eosin (H&E) stained tissue slices (liver, spleen, kidney, heart and lung) of mice injected with hydrogels after 24 h (the white scale bar is 200 µm).
